# Loop-mediated isothermal amplification (LAMP) for point-of-care detection of asymptomatic low-density malaria parasite carriers in Zanzibar

**DOI:** 10.1186/s12936-015-0573-y

**Published:** 2015-01-28

**Authors:** Jackie Cook, Berit Aydin-Schmidt, Iveth J González, David Bell, Elin Edlund, Majda H Nassor, Mwinyi Msellem, Abdullah Ali, Ali K Abass, Andreas Mårtensson, Anders Björkman

**Affiliations:** Department of Microbiology, Tumor and Cell Biology, Karolinska Institutet, SE-171 77 Stockholm, Sweden; Department of Immunology and Infection, London School of Hygiene and Tropical Medicine, London, UK; Foundation for Innovative New Diagnostics (FIND), Geneva, Switzerland; Global Good /Intellectual Ventures Laboratory, Bellevue, WA USA; Zanzibar Malaria Elimination Programme, Ministry of Health, Zanzibar, Tanzania; Global Health (IHCAR), Department of Public Health Sciences, Karolinska Institutet, SE-171 77 Stockholm, Sweden; Centre for Clinical Research Sörmland, Uppsala University, Uppsala, Sweden

**Keywords:** LAMP, Low-density, Malaria, Zanzibar, Elimination, Asymptomatic, Diagnostics

## Abstract

**Background:**

Asymptomatic, low parasite density malaria infections are difficult to detect with currently available point-of-care diagnostics. This study piloted a loop-mediated isothermal amplification (LAMP) kit for field-friendly, high-throughput detection of asymptomatic malaria infections during mass screening and treatment (MSAT) in Zanzibar, a malaria pre-elimination setting.

**Methods:**

Screening took place in three known hotspot areas prior to the short rains in November. Finger-prick blood was taken for screening by rapid diagnostic test (RDT) and LAMP and collected on filter paper for subsequent polymerase chain reaction (PCR) analyses. LAMP results were compared to RDT and to PCR using McNemar’s test.

**Results:**

Approximately 1,000 people were screened. RDT detected ten infections (1.0% (95% CI 0.3-1.6)) whilst both LAMP and PCR detected 18 (1.8% (95% CI 0.9-2.6)) infections. However, PCR identified three infections that LAMP did not detect and *vice versa.* LAMP testing was easy to scale-up in field conditions requiring minimal training and equipment, with results ready one to three hours after screening.

**Conclusions:**

Despite lower than expected prevalence, LAMP detected a higher number of infections than the currently used diagnostic, RDT. LAMP is a field-friendly, sensitive diagnostic test that could be useful for MSAT malaria campaigns which require quick results to enable prompt treatment.

## Background

Asymptomatic and low-density malaria infections are a challenge for areas targeting elimination. Surveillance systems, which are a vital part of malaria elimination efforts, tend to be designed to focus on symptomatic infections captured through routine health systems. However, data suggest that the asymptomatic parasite reservoir can form the majority of infections when malaria transmission is low [1] and contribute substantially to transmission [2,3].

In Zanzibar, where malaria surveillance systems are in place in public health facilities, approximately 1% of febrile patients are confirmed malaria positive (using malaria rapid diagnostic test (RDT) or microscopy) [4]. RDTs have demonstrated their utility in a healthcare settings in Zanzibar [5], where parasite densities are likely to be higher as they have induced symptoms, but community studies suggest they are not effective for detecting asymptomatic, often low-density infections [6]. Molecular tests, such as polymerase chain reaction (PCR) have demonstrated higher sensitivities [7] but are not ideal for point-of-care diagnosis due to the need for advanced laboratory conditions, high cost and relatively long time to results.

Mass screening and treatment (MSAT) is an intervention designed to detect malaria infections in the community. These often asymptomatic and low-density infections are unlikely to present at health facilities and require ultrasensitive, high-throughput, field-friendly point-of-care diagnostic tools to detect them in order to successfully reduce subsequent transmission.

Loop-mediated isothermal amplification (LAMP) offers a field-friendly alternative to PCR. LAMP is less time intensive than PCR and can be performed using heat-blocks, with results read by eye under UV light. It has been successfully developed to detect malaria in a field-stable format [8-10]. The Loopamp™ MALARIA Pan/Pf kit has been trialled in Europe [11,12] and in a health facility setting in Uganda [13], but not in the high-throughput manner required for MSAT. This study, based in Zanzibar, aimed to pilot the kit as the diagnostic tool in a routine MSAT intervention and to assess its practicality and performance for detection of malaria infection compared to RDT and PCR.

## Methods

### Study site and population

Zanzibar is a semi-autonomous archipelago, located 35 km from the coast of mainland Tanzania. Malaria transmission is low, equivalent to a state of pre-elimination. It is seasonal and focal, with the majority of the cases occurring after the long rains in March to May [4]. Short rains occur in November and December. The study took place over 1 week in November 2013 and aimed to screen 1,000 participants from three areas of residual seasonal malaria foci in Unguja island (Panga Tupu, Ukongoroni and Zingwezingwe) where increased malaria incidence had been recorded during the 2013 rainy season.

The communities of the study areas (total population ~1,900) were sensitized to the aims of the study by local chiefs and were asked to report to their local health facility or school for screening on pre-determined dates. All willing participants who reported to the screening points and provided informed consent were tested, regardless of symptoms.

### Sample collection and diagnostics

Screening took place over one or two days in each area. Data collection took place on Nexus 7 tablets using forms designed in Open Data Kit [14]. At screening, blood samples for three different malaria diagnostics were taken from a single finger-prick: 5 μl for RDT (using the collection device provided in the kit), 60 μl for LAMP (using a plastic capillary tube (Dropstir, Medical Precision Plastics, USA)) and approximately 50 μl spotted directly onto filter paper (Whatman 3MM) for PCR.

### RDT

The RDT used in the study was SD Bioline malaria Ag-Pf/pan (Standard Diagnostics Ref 05FK60, Inc; Suwon City, Republic of Korea), targeting *Plasmodium falciparum*-specific histidine-rich protein-2 (HRP-2) and Pan-*Plasmodium* lactate dehydrogenase (pLDH). The test was used in accordance with manufacturer’s instructions. Individuals found positive by RDT were treated using artesunate-amodiaquine, i.e., first-line treatment for uncomplicated malaria, as per national guidelines.

### LAMP

Sixty μl of finger-prick blood was dispensed into a pre-labelled and pre-aliquoted 1.5-ml microtube containing 60 μl of DNA extraction buffer (400 nM NaCl, 40 mM Tris pH 6.5, 0.45 SDS).

### DNA extraction

DNA was extracted by the boil and spin method [13]. Briefly, the tubes containing blood and extraction buffer were vortexed and placed in a heat-block at 95°C for 5 min. The tubes were then centrifuged at 10,000 g for 3 min and 30 μl of the supernatant transferred to a tube containing 345 μl of sterile water.

### Loopamp MALARIA Pan/Pf detection kit

The LAMP assay was performed using Loopamp™ MALARIA Pan Detection Kit (Eiken Chemical Company, Japan) [15]. The Loopamp kit has been described in detail previously [13]. Briefly, 30 μl of diluted DNA extraction was added to each Pan-LAMP tube. Samples were screened in batches of 46 samples (the maximum number of tubes that would fit on the heat-block) and a positive and negative control was included in each run. Following inversion and mixing of the tubes, the strips were placed in a heat-block at 65°C for 40 min, and then transferred for 2 min at 95°C for enzyme inactivation. The results were read immediately by eye under a UV light. All individuals positive for Pan-LAMP were then retested using Pf-LAMP specific kits. Positive individuals (who were not positive by RDT) were informed about their results and given treatment within three hours of screening.

Two heat-blocks (95°C and 65°C), a UV lamp and one centrifuge (24×1.5 ml tubes) were required for LAMP testing. The extraction and the LAMP assays were performed in separate areas to avoid any risk of contamination, however, the heat-block set at 95°C was used for both the DNA extraction and the enzyme inactivation steps. All LAMP tubes were immediately discharged in safety boxes after detection of result. All samples were processed on the same day as sampling.

### PCR

Filter papers were air-dried and packaged in individual sealable bags containing desiccant before being transported to Sweden for PCR analysis. DNA was extracted with the Chelex-100 method using one filter paper punch (3–5 μl) [16]. All samples were screened for parasite DNA with a SYBR Green real-time PCR assay, targeting the Cytochrome b gene of the four major human *Plasmodium* species. The real-time PCR results were analysed by melting curve and gel electrophoresis. PCR products were digested by FspBI enzyme (Thermo Fisher, USA) in RFLP assay for species identification (Weiping Xu, unpublished material). Samples with discordant results between Pan/Pf-LAMP *versus* PCR or RDT were re-extracted in duplicate and each extraction was repeated by PCR in duplicate (=four PCRs). All PCR-positive samples were quantified with an 18S rRNA PCR [17] against standards of known parasite densities.

### Staff training and logistics

Two laboratory technicians employed by Zanzibar Malaria Elimination Programme with no previous experience of LAMP and limited experience of molecular methods were trained for three days to perform DNA extraction and the LAMP assay. Four enumerators were trained to record information on the tablet computers, to take blood and to use and interpret the RDT. Sample collection took place in two health facilities (Zingwezingwe and Ukongoroni) and a school hall (Panga Tupu). DNA extraction and LAMP took place in small rooms within health facilities. LAMP samples from Panga Tupu were transported at ambient temperature 1 km by car to a nearby health facility, equipped with electricity, for processing.

### Data analysis

This was an explorative study to test LAMP in a high-throughput manner as well as determine its sensitivity and specificity. The study aimed to screen 1,000 participants. Based on previous molecular studies in Zanzibar [6], it was estimated that this sample size would detect approximately five RDT positives and up to 40 LAMP positives. Data were downloaded from the Nexus 7 tablets into STATA v12 (Statacorp, Texas, USA). The diagnostic performance was assessed by calculating the sensitivity and specificity of RDT and LAMP using Cytochrome-b PCR as the reference standard and assessed using McNemar’s test.

### Ethical issues

Written informed consent was obtained from all participants or guardians. Ethical approval was obtained from the Zanzibar Medical Ethical Committee (ZAMEC) (ZAMREC/0001/September/013) and from the Regional Ethics Review Board, Stockholm, Sweden (2013/836-32).

## Results

### Study population

Approximately 250 samples were processed per day. A total of 996 samples had matching RDT, LAMP and PCR results: 271, 422 and 303 from Panga Tupu, Ukongoroni and Zingwezingwe, respectively. This represented 81, 51 and 41% of the estimated populations of each area. The median age of participants was 12 years old (range: one month to 95 years) and 54% of people screened were female.

### Practicality of sample collection and processing of LAMP

On the whole, the process was simple and the enumerators and lab technicians experienced few problems. Some difficulty was experienced using the plastic capillary tube and obtaining the full volume of blood required, however, the enumerators were able to improve their technique with practice. The DNA extraction and LAMP test were simple to perform and no bottlenecks were experienced at any stage of the process, although a higher number of samples per day would have been difficult to process with just one LAMP station. The readings of LAMP results via UV light were easy to perform with good inter-observer agreement. All Pan-LAMP results were available within 3 hours of sample collection. Pf-LAMP was run on all Pan-LAMP positives at the end of each day.

### Malaria indices

**Figure 1:**
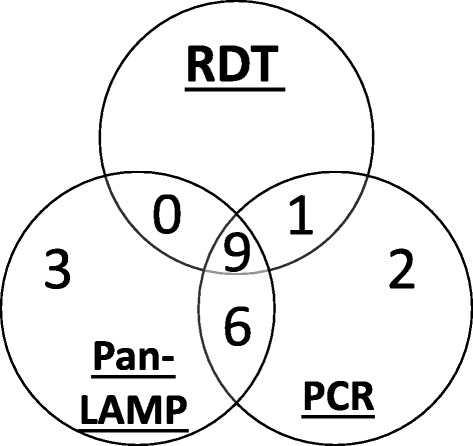
**Venn diagram showing the distribution of positive RDT, Pan-LAMP and PCR results.**

Overall 10/997 (1.0% (95% CI, 0.1-8.1%) individuals were RDT positive, whereas both Pan-LAMP and *cyt-b* PCR detected 18/997 (1.8% (0.2-18.0%) malaria infections (Table 1). All RDT positives were confirmed by PCR, but one RDT positive was not positive by LAMP (Figure 1). Three of the Pan-LAMP positive samples were not confirmed by PCR. Nine of the Pan-LAMP positive samples were also positive for Pf-LAMP (Table 1).

**Table 1:** **Results, sensitivity and specificity of LAMP (Pan and** ***Plasmodium falciparum*** **) and RDT by screening site**

**Method**	**Positive no.**	**Negative no.**	**Sensitivity, % (95% CI)**	**Specificity, % (95% CI)**
***Total***				
**PCR**	18 (15 Pf, 1 Pm, 2 undetermined)	978	Reference	
**Pan-LAMP**	18	978	83.3 (58.6-96.4)	99.7 (99.1-99.9)
**Pf-LAMP** ^**¤**^	9	9	60.0 (32.3-83.7)	
**RDT**	10	996	55.6 (30.8-78.5)	100 (99.6-100)
***Panga Tupu***				
**PCR**	11 (9 Pf, 2 undetermined)	260	Reference	
**Pan-LAMP**	11*	260	81.8 (48.2-97.7)	99.2 (97.2-99.9)
**Pf-LAMP** ^**¤**^	6	5	66.7 (29.9-92.5)	
**RDT**	5	266	45.5 (16.7-76.6)	100 (98.6-100)
***Ukongoroni***				
**PCR**	1 (Pf)	421	Reference	
**Pan-LAMP**	2	420	100 (2.5-100)	99.8 (98.7-100)
**Pf-LAMP** ^**¤**^	1	1	100 (2.5-100)	
**RDT**	1	421	100 (2.5-100)	100 (99.1-100)
***Zingwezingwe***				
**PCR**	6 (5 Pf, 1Pm)	297	Reference	
**Pan-LAMP**	5**	298	83.3 (35.9-99.6)	100 (98.8-100)
**Pf-LAMP** ^**¤**^	2	4	40.0 (5.3-85.3)	
**RDT**	4	299	66.7 (22.3-95.7)	100 (98.8-100)

*2 not confirmed by PCR (Pf-LAMP negative).

**1 not confirmed by PCR (Pf-LAMP negative).

^¤^Pf LAMP only performed on Pan-LAMP positives so true specificity cannot be determined.

Using PCR as the reference standard, RDT detected approximately half of the infections, with a sensitivity of 55.6% (30.8-78.5%) whilst Pan-LAMP detected approximately 4/5ths of the PCR determined infections with a corresponding LAMP sensitivity of 83.3% (58.6-96.4%) (McNemar’s test: p = 0.06). Both RDT and LAMP had specificities over 99% against PCR.

Of 18, 15 (83%) of the PCR positive samples were *P. falciparum*, one was *Plasmodium malariae*, and the remaining two had unsuccessful species determination (Table 2). The real-time PCR determined *P. falciparum* geometric mean density was 26 parasites/μl (range: 0–4,626). The corresponding mean parasite densities were 47 parasites/μl (range 0–4,626) and 661 parasites/μl (range 2–4,626), respectively, for Pan-LAMP and RDT positive samples. The three Pf-LAMP-negative but PCR-positive *P. falciparum* infections had parasite densities of 3, 24 and 293 parasites/μl, all these were, however, positive by Pan-LAMP and the highest density (293 parasites/μl) was also positive by RDT.

**Table 2:** **Results for all Cytochrome b real-time PCR positive samples**

**ID**	**RDT result**	**LAMP Pan**	**LAMP Pf**	**RFLP**	**Parasites (per μl blood)**
1	-	+	-	*P. malariae*	0
2	-	-	N/D	*P. falciparum*	1
3	-	-	N/D	*P. falciparum*	2
4	-	+	-	*P. falciparum*	3
5	-	+	-	N/D	3
6	-	+	+	*P. falciparum*	5
7	-	+	-	N/D	7
8	-	+	+	*P. falciparum*	12
9	-	+	-	*P. falciparum*	24
10	-	+	+	*P. falciparum*	119
11	+	-	N/D	*P. falciparum*	2
12	+	+	+	*P. falciparum*	9
13	+	+	+	*P. falciparum*	17
14	+	+	+	*P. falciparum*	176
15	+	+	-	*P. falciparum*	293
16	+	+	+	*P. falciparum*	311
17	+	+	+	*P. falciparum*	1169
18	+	+	+	*P. falciparum*	4626

N/D: Not determined.

## Discussion

Highly sensitive, field-friendly diagnostics are required to enable prompt detection and treatment of low-density malaria infections during routine MSAT in low transmission areas. The Loopamp™ MALARIA Pan Detection Kit was simple to scale up for relatively high-throughput screening in a field setting, requiring just three days of training for staff with no previous experience of LAMP and limited experience of molecular methods. Results were available within one to three hours of screening and showed similar high sensitivities as were demonstrated in Uganda and in reference laboratories in Europe [11,18]. However, if LAMP is going to be a useful tool in routine MSAT interventions in areas aiming at malaria elimination, there is a need for even higher throughput (i.e. fewer transfer steps, ability to run more samples simultaneously) for screening of larger numbers of samples.

Pan-LAMP detected three positive samples that were not confirmed using PCR. This is not uncommon with a test that has a similar detection threshold as the reference standard and was also seen in a previous study in Uganda [13]. Previous studies have questioned whether this is due to false-positive LAMP or chance discrepancy due to the low parasite density [11]. Conversely, three PCR-positive infections were not confirmed using Pan-LAMP. Importantly, these three samples all had parasite densities lower than five parasites/μl. In addition, the Pf-LAMP test did not detect several *P. falciparum* infections detected by Pan-LAMP, perhaps due to a lower detection limit (5 *versus* 7.5 DNA copies/test) [12], although one sample had approximately 300 parasites/μl. With such low numbers of positive samples it is difficult to make any conclusions about the Pf-LAMP, however in Zanzibar, with a majority of infections being either *P. falciparum* and/or *P. malariae* it may be pertinent to solely use Pan-LAMP for screening purposes during MSAT.

The prevalence of low density infections in the screening areas were lower than expected, based on previous molecular studies in Zanzibar [6], which have detected up to an eight-fold higher prevalence using PCR compared to RDT/microscopy. The study sites were selected based on higher than average incidence of symptomatic cases reported to health facilities during the long rains in March to May 2013. This highlights the complex spatial and temporal dynamics of hotspots of transmission in a malaria pre-elimination setting. Despite the low prevalence, there was some evidence that LAMP detected a higher proportion of infections present than the currently used tool. It is likely that if prevalence had been higher, this result would have statistically significant and in epidemiological terms, it remains important. The use of LAMP during MSAT is likely to result in a larger number of infections being treated, however, whether this will result in lowered transmission is still to be fully evaluated.

## Conclusion

Currently, the Loopamp™ MALARIA Pan Detection Kit is more expensive and less simple than RDT and thus unlikely to be rolled out for use in health facilities in Zanzibar, where malaria RDT is a reasonably cost-effective tool to detect symptomatic, generally higher parasite density, infections. However, the need for more sensitive diagnostics during routine MSAT interventions in low-endemic settings is clear. LAMP is a simple, sensitive, field-friendly diagnostic which can be performed outside of laboratory conditions and which could play an important role in MSAT for improved detection and treatment of low-density parasitaemias in order to reduce post-intervention malaria transmission.
